# Donatio organorum- reluctance to organ donation medical students; a cross-sectional descriptive study

**DOI:** 10.1016/j.amsu.2022.104534

**Published:** 2022-09-30

**Authors:** Sumia Fatima, Zainab Hussain, Sidra Hamid, Zainab Idrees, Maryam Mansoor, Tayyaba Idrees

**Affiliations:** a 4^th^ Year MBBS Student at Rawalpindi Medical University, Rawalpindi, Punjab, Pakistan; b Department of Physiology at Rawalpindi Medical University, Rawalpindi, Punjab, Pakistan

**Keywords:** Organ donation, Organ transplantation, Tissue and organ procurement (D009927), Medical students (D013337), Willingness, Awareness (D001364), Knowledge (D019359)

## Abstract

**Introduction:**

Organ donation is the act of removing an organ or tissue from a donor and transplanting the said organ/tissue to a recipient. Despite being the only definitive treatment for end-organ failure, there is a lot of hesitation surrounding the practice of organ donation. Even among medical students, who are more aware of the benefits of organ donation than the general public, this reluctance is widespread.

Therefore, we conducted this research to determine the basis of this skepticism, so that the root causes can be identified and eradicated. Our research sought to ascertain the overall attitudes of Rawalpindi Medical University, Pakistan's students towards organ donation, the associated factors that influenced this attitude (religious devotion, gender, age, year of study) and the reasons for the hesitance.

**Methods:**

A cross-sectional study targeting the students of first to final year MBBS was conducted at Rawalpindi Medical University, Pakistan in the year 2022. The sample size was calculated using OpenEpi software, and came out to be 292. The students enrolled at Rawalpindi Medical University during the year 2021–2022 were made a part of the study. A self-structured questionnaire that was developed after substantial research was used to collect the data using a non-random convenience sample technique. Chi Square test was used to determine significance after data analysis using SPSS-22.

**Results:**

A total of 290 students participated in the study, 58 from each year. All of the participants were Muslim. A very strong correlation was found between high devoutness and willingness towards organ donation (p = 7.4252E-13). Only 9/290 (3%) of people in Pakistan have joined The Transplantation Society of Pakistan; the main cause of this low ratio is that very few people were aware that such a group even existed (according to 62% of the responders). The mistrust of doctors and the belief that appropriate efforts would not be done to resuscitate patients who have signed up for organ donation is a significant factor in the anxiety surrounding organ donation.

**Conclusions:**

There are several reasons why people are reluctant to donate their organs, including a lack of understanding of religious perspectives on the subject, mistrust of medical professionals and medical administration, and general public ignorance. We can make a significant progress toward closing the gap between the demand for and supply of organ donations if these problems are remedied. The most effective strategy to stop organ trafficking is through organ donation. Through seminars, conversations, and workshops, we need to raise awareness about organ donation.

## Highlights

• We found a positive correlation between religious devoutness of Muslim students and willingness to donate.
• The main reason for reluctance was the fear of being premature pronounced dead in the hope of harvesting their organs.
• Only 3% of the study participants had joined the transplantation society of Pakistan.
• The most effective strategy to stop organ trafficking is through organ donation.

## 1 Introduction

Organ donation is the procedure of surgically removing an organ or tissue from one individual (the organ donor) and inserting it into a new individual (the recipient) [1]. It is the only treatment for end-stage organ failure that is now available and can prolong and enhance human life [2]. There still remains a serious scarcity of suitable organs for transplantation despite the fact that organ donation is seen as a major necessity and that substantial support has been given in this area globally [3]. 6000 patients die every year while waiting for an organ due to this shortage [4]. This problem is comparable much worse in a country like Pakistan, where organ failure claims the lives of 50,000 people annually. Statistics show that just 35.3% of Pakistan's population is willing to donate an organ, which drives up the need for donations even more [5,6]. Numerous psycho-social factors, including education, religion, social influence, and moral standards, have an impact on this attitude regarding organ donation. Of these, religious views stand out as the most significant psycho-social element [7,8]. Several bioethical difficulties, such as organ trafficking, organ harvesting and transplantation tourism flourishing in economically underdeveloped nations, are further causes for this hesitation [9]. These statistics unequivocally demonstrate the significance of organ donation; therefore effective schemes are needed to be formulated to decrease the hesitancy among general public. This idea can be promoted by increasing knowledge and awareness; in which medical students can play a significant part in changing people's perceptions of organ donation for the better [10,11] (see Table 1).

**Table 1:** Relation between religious devoutness and willingess towards organ donation.

		How Much Are You Willing To Donate Organs After Death?					Tota l
		Certainly not	Certainly yes	Maybe yes, Maybe not	Probably not	Probably yes	
Are You A Staunch Believer And Practice Your Religion Devotedly?	Maybe, I try my best	0	0	80	23	28	131
	to pray daily but skip a						
	few prayers and I am						
	not regular in reciting						
	the Holy Book and						
	other stuff						
	No, I believe in my	4	8	10	5	0	27
	Religion and its						
	principles but I fail to						
	follow them through						
	Yes, I am a staunch	21	20	45	9	37	132
	believer. I pray daily,						
	recite my Holy Book						
	and gain Religious						
	Knowledge.						
Total		25	28	135	37	65	290

In light of this, we set out to ascertain the attitudes of the medical students at Rawalpindi Medical University, Pakistan towards organ donation, the correlation

between the prevalent attitudes and various factors like religious adherence, gender, age, and year of study, as well as the primary causes of the hesitation towards organ donation.

## 2 Methodology

**4(A)** Registration was done in the research directory of the university Rawalpindi Medical University, Pakistan. After gaining informed consent, data were collected from medical students using self-structured questionnaire. Participants' confidentiality and identities were upheld.

**4(B)** The study's synopsis was submitted to the Rawalpindi Medical University's Ethical Review Board and was granted ethical approval; however, ethical approval is not necessary for this study specifically as it does not involve any interventions, medical or surgical, or other research that could negatively impact the study participants' health (mental or physical).

**5(A)** This study is a cross-sectional descriptive study.

**5(B)** It was conducted at Rawalpindi Medical University, Pakistan (a public sector medical university) during the year 2022.

**6(A)** Students of first year to final year made up the study population. The students who were enrolled in Rawalpindi Medical University's first, second, third, fourth, or final year of MBBS during the academic year 2021–2022 met the inclusion criteria. Participants who were unwilling to engage in the study and openly declare their religious convictions were excluded from it as an exclusion criterion.

**6(C)** Sample size was calculated using the OpenEpi software as follows; Population size (for finite population correction factor or fpc)(N): 1200.

Hypothesized % frequency of outcome factor in the population (p): 50% ± 5.

Confidence limits as % of 100(absolute +/- %)(d): 5% Design effect (for cluster surveys-DEFF): 1.

### 2.1 Sample size

95% 292.

### 2.2 Equation

Sample size n = [DEFF*Np(1-p)]/[(d2/Z21-α/2*(N-1)+p*(1-p)]

The technique utilised for data collection was non-random convenience sampling.

After conducting a thorough literature analysis and researching this subject from the perspectives of many religions, a self-structured questionnaire was created to collect data. Guidelines were also taken from NHS London videos on barriers regarding organ donation. Additionally, cultural values were taken into account.

Questionnaire consisted of an Introduction paragraph outlining the purpose of research and taking consent of the participant.

The following section gathered data on the participant's gender, religion, and academic year.

The final section included 15 questions that used the Liekert scale to gauge the attitude of participants towards organ donation.

**9** IBM SPSS Statistics −22 was used to analyse the data, and the chi square test was used to determine the relationship between the various variables.

The work has been reported in line with the STROCSS criteria [12].

## 3 Results

The results obtained were:

290 students from the first to fifth year of MBBS willingly participated in the study, 58 from each year. Half were males and half females.

All the students were Muslims and majority 45.5% (n = 132/290) were devout Muslims who offered daily prayers and recited the Holy Quran, 45.2% (n = 132/290) were irregular in their prayers and in reciting Holy Quran while 9.3% (n = 27/290) claimed to believe in Islam but did not follow its teachings.

Religious values and readiness to donate organs have a substantial relationship (p = 7.4252E-13, where *a* is 0.05).

The majority of students 60% (n = 174/290) believed organ donation after death is an appreciable act, 38.6% (n = 112/290) were uncertain whereas 1.4% (n = 4/290) thought that it was not.

There was also a strong link between thinking that donating organs is a commen-dable act and readiness to do so (p = 1.3785E-17).

**Fig. 1:**
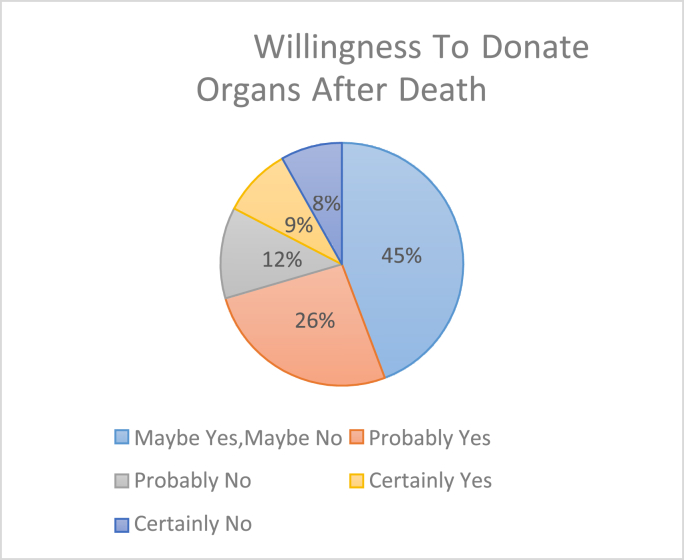
Willingness to donate organs after death.

Fig. 1 indicates the students’ willingness to donate their organs.

**Fig. 2:**
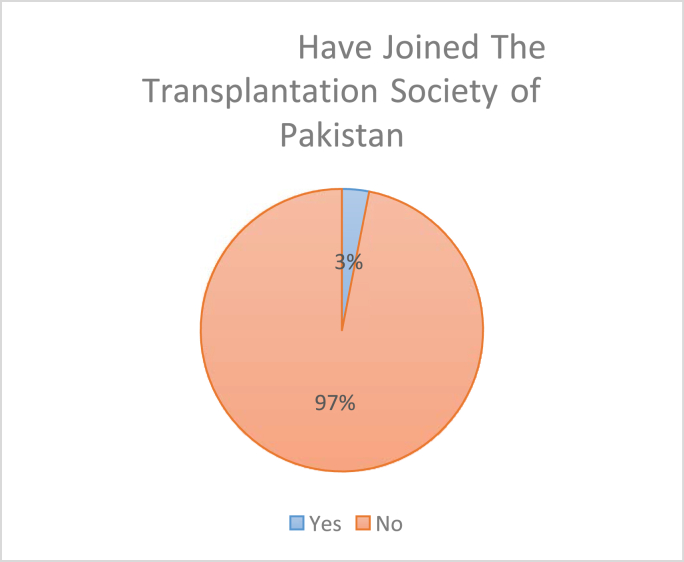
Have joined the transplantation society of Pakistan.

Shockingly, only 9 out of 290 (3.1%) responders have joined The Transplantation society of Pakistan whereas 281 (96.9%) have not joined any society (Fig. 2).

33.4% (n = 97/290) were unsure whether to join the transplantation society, 9.3% (n = 27) were certain to join, and 10.3%

(n = 30) were certain not to join, 27.6% (n = 80) said they are likely to join, and 19.4% (n = 56) said they are unlikely to join.

**Fig. 3:**
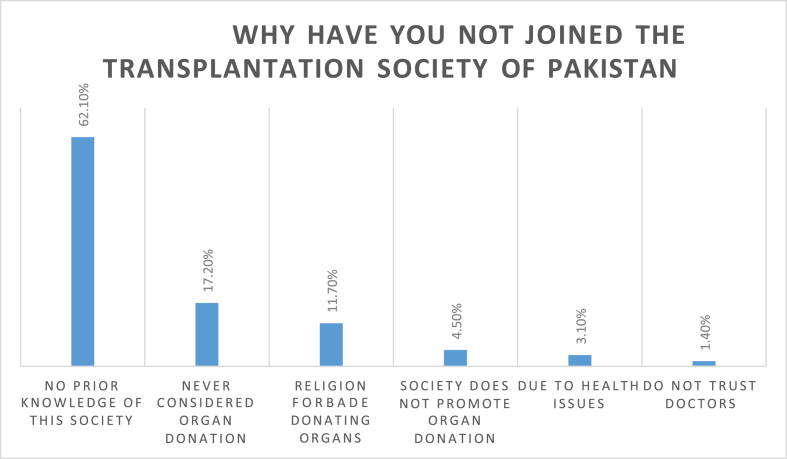
WHY have YOU not joined the transplantation society of Pakistan.

When asked the reason for not joining the Transplantation Society of Pakistan, around two-thirds (62.1%) responded that they had no prior knowledge of such an organisation. The rest of the responses are depicted in Fig. 3.

The majority of respondents (74.4%) were in favour of forming an ethical committee to ensure that doctors do not do immoral or unethical acts such as deliberately failing to preserve a patient's life in the hope of harvesting their organs.

219 out of 290(75.5%) have not discussed their willingness or unwillingness to donate organs with their family, 57(19.7%) have discussed with family while 14(4.8%) may have discussed.

There is a substantial link between willingness to donate organs and having had a discussion with family (p = 1.1697E-

9), with those who have not discussed being unsure whether they want to donate.

38.3% (111/290) believed that the practice of procuring organs of hanged convicts should become prevalent and money should be paid to family of prisoner and victim. 31.0% (n = 90) did not believe in this practice while 30.7% (n = 89) said they were unsure.

49.0% (n = 142/290) believed that illegal trade of organs would be reduced if organ donation becomes common, 20.3% (n = 59) did not think like that whereas 30.7% (n = 89) were uncertain. Majority 74.5% (216/290) believed that increasing public knowledge would enhance people's willingness to donate organs.

There is no link of year of study (p = 0.083) or gender (p = 0.87) with the willingness to organ donation.

## 4 Discussion

In Islam there are a number of fatawa (religious edicts) with regards to organ donation [[13], [14], [15]].

Islamic scholars and imams play an important role in providing guidance to members of the Muslim community on organ donation.

Over 100 fatawa (religious edicts) have been produced around the world about organ donation and three of these have been published in the UK. Nearly all Islamic scholars unanimously agree upon the topic of living organ donation, "It is permissible for a living person to donate part of the body such as the kidneys to save the life of another, provided that the organ donated would not endanger the donor's life and that it might help the recipient and also it is not done for monetary reasons." [16,17].

In June 2019, a UK-based Sunni scholar, Mufti Mohammed Zubair Butt, a Juris consult from the Institute of Islamic Jurisprudence in Bradford produced a fatwa, “Organ Donation and Transplantation in Islam”.

“Once it is categorically determined that the person is dead, then the cessation of the heart and the lungs is irreversible and after that donation can take place” [18].

In 2000, the European Council for Fatwa and Research (ECFR), based in Dublin, Ireland, declared its ratification of the resolutions of both the Islamic Fiqh Academy (IFA) of the Muslim World League and the International Islamic Fiqh Academy (IIFA) and declared organ donation after death permissible [19].

The basic position of the 1995 fatwa of the Muslim Law (Shariah) Council was that organ transplantation is permissible, and brain-stem death is a proper definition of death.

Organ donation is also allowed in Christianity [20], Judaism [21], Hinduism [22], and even Buddhism [23].

Because of the contradictory statements of different scholars on organ donation [24], the awareness of the people regarding organ donation [1,4]and different related societies working in Pakistan like the Transplantation Society of Pakistan.

[25] is very less. As shown by our research, people think that it is a commendable act to donate organs after death but willingness to donate is less, and active joining

of the Transplantation Society is even lesser. Discussions about organ donation with families are considered a near-taboo.

Aside from Religious reasons, another important reason that hampers the attitude towards organ donation is people's distrust in doctors and medical administration. People think that they would not get proper medical treatment if they were designated organ donors [26].

“*Doctors would not try as hard to save me if they knew I was an organ donor*” [27].

Also, people believe that "selected people" would receive donated organs, not those who needed them the most [28]. To counter this fear, many countries like America have made ethical committees that ensure that the physician's primary concern be the health of the patient. The death of the donor shall have been determined by at least one physician other than the recipient's physician. Death shall be determined by the clinical judgment of the physician, who should rely on currently accepted and available scientific tests and other detailed rules set by the committee to ensure ethical transplantation practice [28].

The students stated their mistrust of the medical professionals regarding organ donation because they are fully aware of the system's shortcomings as members of the healthcare system. The medical students enter the university young, naïve, and ready to learn with full optimism in their hearts but by the time they are in their last year, their perspectives have completely changed [29]. It's unclear why their views change, but they could be related to a ceiling of high attitude scores at entry, a decline in idealism, or the effects of the unexpected curriculum [30].The empathy levels of the doctors fall through the years. Three longitudinal and six cross-sectional studies of medical students in a systemic review done demonstrated a significant decrease in empathy during medical school [31].

Doctors have been involved in illegal organ donation. A doctor was held accountable in Germany for altering at least 25 patient files to move them up the transplant waiting list, endangering the lives of those who were most in need [32]. There is a significant underground market for organ donation in several nations, thus some doctors also do it for financial gain [33].

Organ Donation is considered to be the only way to curb Organ Trafficking [34]. The trafficking of human beings for the purpose of organ removal (THBOR) is not a new phenomenon [35]. With a shortage of legally sourced organs around the world, it is estimated that the illegal trade of human organs generates about 1.5

billion dollars each year from roughly 12,000 illegal transplants. As shown by our research, the majority believes that if organ donation becomes common, illegal trades of organs can be reduced [35].

Majority responded in our research that organ donation from hanged convicts should become common and the money should be paid to the victims and the convict's family. Two-thirds of organ donors in Chinaare executed prisoners, state media reported [36] World Medical Association agreement – signed by China among others – asks countries not to use organs from death-row prisoners because of concerns about whether they have truly given informed consent [37]. But some researches have shown that the prisoners themselves want to donate organs to atone for their sins, as quoting one prisoner:

“*There is no way to atone for my crimes, but I believe that a profound benefit to society can come from my circumstances. I have asked to terminate my remaining appeals, and then donate my organs after my execution to those in need*” [38].

So if proper way can be made to obtain the consent of the prisoners, this issue can be resolved.

## 5 Strengths and limitations

This study will contribute to illuminating some of the key causes of hesitancy regarding organ donation. Better understanding of Islam was discovered to be a factor in favour of organ donation, which was rather a surprising outcome from our study. Thus, our research will aid in reducing some of the unfavourable stereotypes about Muslims. However, because this was a study from just one institute, much more research is needed before the findings can be applied generally.

## 6 Relevance and implications

Our study demonstrates that, even among the more educated segments of society, there is a dialogue about organ donation that has to be had. According to our study, medical students in particular, who are supposed to be much more accepting of organ donation, did not exhibit a particularly positive attitude. Further actions can be done to promote organ donation, primarily through increasing registrations with The Transplantation Society of Pakistan, after a more comprehensive survey that includes all of Pakistan's medical colleges provides a clearer picture of the current situation.

## 7 Conclusion

There are several reasons why people are reluctant to donate their organs, including a lack of understanding of religious perspectives on the subject, mistrust of medical professionals and medical administration, and general public ignorance. We can make a significant progress toward closing the gap between the demand for and supply of organ donations if these problems are remedied. The most

effective strategy to stop organ trafficking is through organ donation. Through seminars, conversations, and workshops, we need to raise awareness about organ donation.

## Funding

None.

## Provenance and peer review

Not commissioned, externally peer-reviewed.

## Ethical approval

The ethical approval form was acquired from the Ethical Review Board of the Rawalpindi Medical University signed by the Dean of Community Medicine and Public Health Prof. Dr Syed Arshad Sabir and Assistant Professor of Community Medicine and Public Health. It was a descriptive study for academic purposes not using any interventional procedures. Consent of the participants was taken in the first section of the questionnaire. The confidentiality of the participants was ensured.

## Please state any sources of funding for your research

None.

## Author contribution

Sumia Fatima (4th year MBBS student): study conception and design and discussion data collection.

Zainab Hussain (4th year MBBS Student): data analysis.

Dr Sidra Hamid (Assistant Professor of the Department of Physiology) Supervisor and Final Review and approval.

Zainab Idrees (4th year MBBS): Methodology.

Maryam Mansoor (4th year MBBS): Introduction.

Tayyaba Idrees (4th year MBBS) Compiling and Editing (corresponding author)

## Registration of research studies

1. Name of the registry: Student Registry of the University Rawalpindi Medical University.

2. Unique Identifying number or registration ID:

As it is a simple cross sectional study, not any interventional study or clinical trial, so it is not registered in any public accessible registry. No intervention (medical or surgical) was done in this study.

## Guarantor

Sumia Fatima: 1st author 4th year MBBS Rawalpindi Medical University Pakistan sumiahfatima3@gmail.com +92 3319299897.

You can contact me on this email or WhatsApp number to acquire any data.

## Consent

The section one of the Questionnaires requested for written consent of the participants. The research is for academic purpose only. Confidentiality of the participants was maintained. Names, registration numbers or other details revealing IDs were not inquired from the patients.

## Declaration of competing interest

The authors report no conflicts of interest.
